# A Novel Unitary ESPRIT Algorithm for Monostatic FDA-MIMO Radar

**DOI:** 10.3390/s20030827

**Published:** 2020-02-04

**Authors:** Feilong Liu, Xianpeng Wang, Mengxing Huang, Liangtian Wan, Huafei Wang, Bin Zhang

**Affiliations:** 1 State Key Laboratory of Marine Resource Utilization in South China Sea and School of Information and Communication Engineering, Hainan University, Haikou 570228, China; feilongliu@hainu.edu.cn (F.L.); wong9525@163.com (H.W.); 2 Key Laboratory for Ubiquitous Network and Service Software of Liaoning Province, School of Software, Dalian University of Technology, Dalian 116620, China; wanliangtian@dlut.edu.cn; 3 Department of Mechanical Engineering, Kanagawa University, 3-27-1 Rokkakubashi, Kanagawa-ku, Yokohama 221-8686, Japan; zhangbin@kanagawa-u.ac.jp

**Keywords:** Unitary ESPRIT, FDA-MIMO radar, parameter estimation, phase period ambiguity

## Abstract

A novel unitary estimation of signal parameters via rotational invariance techniques (ESPRIT) algorithm, for the joint direction of arrival (DOA) and range estimation in a monostatic multiple-input multiple-output (MIMO) radar with a frequency diverse array (FDA), is proposed. Firstly, by utilizing the property of Centro-Hermitian of the received data, the extended real-valued data is constructed to improve estimation accuracy and reduce computational complexity via unitary transformation. Then, to avoid the coupling between the angle and range in the transmitting array steering vector, the DOA is estimated by using the rotation invariance of the receiving subarrays. Thereafter, an automatic pairing method is applied to estimate the range of the target. Since phase ambiguity is caused by the phase periodicity of the transmitting array steering vector, a removal method of phase ambiguity is proposed. Finally, the expression of Cramér–Rao Bound (CRB) is derived and the computational complexity of the proposed algorithm is compared with the ESPRIT algorithm. The effectiveness of the proposed algorithm is verified by simulation results.

## 1. Introduction

The multiple-input multiple-output (MIMO) radar [1,2], which utilizes multiple antennas to simultaneously transmit diverse waveforms and receive the reflected signals in similar ways, has many potential advantages [3]. Unlike the conventional phased-array (PA) radar, MIMO radar has many superiorities based on its spatial diversity and waveform diversity, such as improving the system performance with higher degrees-of-freedom (DOFs) [4,5]. Normally, MIMO radar can be classified into two types based on the spatial location of antenna elements. One is named distributed MIMO radar, where the transmitting or receiving array elements are placed in different positions, with a relatively large spacing of elements [6]. The other is the collocated MIMO radar, where both transmitting and receiving antennas are arranged close to each other [7]. According to whether the receiver and transmitter are located in the same place, the collocated MIMO radar is further categorized into the monostatic MIMO radar [8,9] and bistatic MIMO radar [10,11]. A monostatic MIMO radar is superior in its excellent maneuverability and synchronization between the transmitter and receiver. In the contemporary defense system, the monostatic radar system is the most mainstream and common sensor unit in the modern radar network system. A monostatic MIMO radar is addressed in this paper.

In radar systems, the target localization is one of the main issues. The angle and range estimation are the important parts for target localization [12,13,14,15,16,17,18,19]. However, in the beam scanning of both the PA radar and MIMO radar, the beam pointing is angle-dependent and range-independent. The frequency diverse array (FDA) radar, in which the direction of focus changes with range, is proposed in [20]. For the FDA radar, the frequencies of each transmitting array element are different, which can lead to a range-angle-dependent beampattern [21]. Then, joint angle and range can be estimated simultaneously in the FDA radar [22,23,24]. Nevertheless, the resolution of parameter estimation is influenced by the maximum frequency increment and the array aperture [25]. The maximum number of distinguishable targets is determined by the number of DOFs and the frequency increment. The range-dependent beampattern produced by a linear frequency modulation structure is analyzed [26]. In order to estimate angle and range, a double-pulse method was introduced to obtain the range and angle separately, where the antenna successively transmits two pulses with non-zero and zero frequency increments [27]. A transmitting subaperture scheme in FDA radar was investigated, where the uniform linear array (ULA) is divided into multiple overlapping subarrays [28]. To decouple the angle and range, a special FDA radar with different frequency increments was proposed [29]. A method based on nonuniform frequency increment was proposed to decouple the FDA beampattern [30]. There are some other interesting investigations reported in [31,32,33,34] about decoupling range and angle in the FDA radar.

In the last few years, FDA-MIMO radar was regarded as a novel radar system combining the advantages of FDA radar with MIMO radar [35,36,37]. The FDA-MIMO radar can decouple angle and range by exploiting the high DOFs of MIMO technique and the angle-range-dependent beampattern of FDA radar [28,37]. Furthermore, Xu et al. applied a maximum likelihood estimator to obtain an unambiguous angle and range estimation [38]. The sparse reconstruction-based algorithm was utilized for the target location with an FDA-MIMO radar [39]. Multiple signal classification (MUSIC) algorithms were also introduced to the FDA-MIMO radar [40,41]. However, these algorithms with peak-searching require a large computational complexity. Although a method based on estimation of signal parameters via rotational invariance techniques (ESPRIT) algorithm has been introduced to obtain the range and angle estimation and reduce the complexity [42], there are still some challenges, such as a low estimation performance, high complexity and phase ambiguity.

In this paper, an improved unitary ESPRIT method for angle and range estimation in monostatic FDA-MIMO radar is presented. Then, a method is proposed to achieve the automatic pairing of angle and range. The reason for phase ambiguity is analyzed, and a simple and efficient solution is proposed. The Cramér–Rao Bound (CRB) for target parameter of monostatic FDA-MIMO radar is derived. Computational complexity analysis is provided to be compared with that of [42].

The paper is summarized as follows. Section 2 introduces the signal model of the monostatic FDA radar. We propose an improved unitary ESPRIT algorithm with an automatic pairing, and investigate a phase judgment in Section 3. In Section 4, we derive the CRB of the monostatic FDA-MIMO radar and offer the specific analysis of computational complexity. Several simulation results are provided to indicate the effectiveness of the presented algorithm in Section 5. The conclusion of the paper is indicated in Section 6.

## 2. Signal Model of Monostatic FDA-MIMO Radar

**Figure 1:**
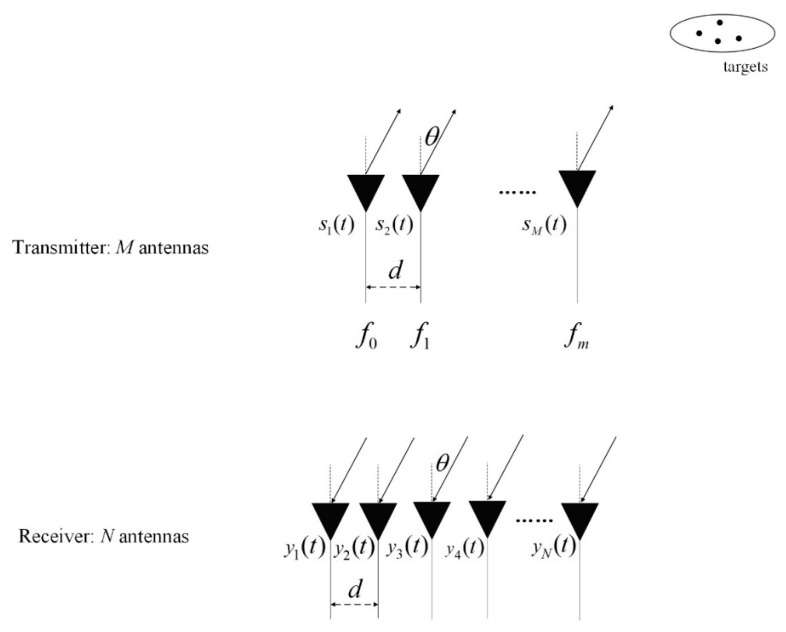
Monostatic FDA-MIMO radar.

The signal model of monostatic FDA-MIMO radar is shown in Figure 1, which is configured with *M*-antenna-transmitting ULA and *N*-antenna-receiving ULA. The transmitting and receiving arrays are placed together to guarantee that the DOA is the same as the direction of departure (DOD). The element interval of the transmitting array is equal to that of receiving array, and they are both marked with *d*. The element interval *d* is set to half of the maximal wavelength. The first element of transmitting array is assumed as the reference. The frequency of the *m*-th signal emitted by the transmitting array is calculated by

(1) $$f_{m}=f_{0}+(m-1)\Delta f,\;m=1,2,\cdots ,m$$

where *f*_0_ is the referenced frequency, and Δ*f* is the deviation of adjacent transmitting frequencies, where Δ*f* << *f*_0_. The baseband signals emitted by the different elements are mutually orthogonal. Assume that the emitted signal of *m*-th element satisfies

(2) $$s_{m}(t)=\sqrt{\frac{E}{M}}\phi_{m}(t)e^{j2\pi f_{m}t},\;0\leq t\leq t$$

where *E* is the total transmitted energy, *T* is the radar pulse duration, and $\phi_{m}(t)$ denotes the baseband waveform. Suppose that all baseband signals are orthogonal to each other and normalized

(3) $$\int_{T}\phi_{m}(t)\phi_{n}^{*}(t-\tau )e^{j2\pi (m-n)\Delta ft}dt=\{\begin{array}{c} 0,\\ 1, \end{array}\begin{array}{c} \;m\neq n,\\ \;m=n \end{array}\begin{array}{c} \forall \tau \end{array}$$

where (.)* stands for the conjugate operator and *τ* is the time delay. There are *K*-independent targets distributed in the far-field, and the ranges of all targets are much larger than the FDA-MIMO radar aperture. The angle and range of *k*-th target are represented by (*θ_k_*, *r_k_*). Due to the limitation of the maximum unambiguity range, *r_k_* should be less than *c*/(2Δ*f*), and *c* denotes the speed of signal propagation, and *c*/(2Δ*f*) represents the maximum unambiguity range [32]. The expression of steering vector of transmitting array is [33]

(4) $$a(r_{k},\theta_{k})=r(r_{k})⊙d(\theta_{k})\in {\mathbb{C}}^{M\times 1}$$

(5) $$r(r_{k})=[1,e^{-j4\pi \Delta fr_{k}/c},\cdots ,e^{-j(m-1)4\pi \Delta fr_{k}/c}{]}^{T}\in {\mathbb{C}}^{M\times 1}$$

(6) $$d(\theta_{k})=[1,e^{j\pi \sin\theta_{k}},\cdots ,e^{j(m-1)\pi \sin\theta_{k}}{]}^{T}\in {\mathbb{C}}^{M\times 1}$$

where *r*(*r_k_*) and *d*(*θ_k_*) denote the range-dependent part and the angle-dependent part of transmitting the joint-steering vector of the *k*-th target, respectively. ⊙ stands for Hadamard product operator. (.)^T^ denotes the transpose operator. The coupling relationship between angle and range can be shown in Equation (4). The receiving spatial steering vector can be given by [34]

(7) $$b(\theta_{k})=[1,e^{j\pi \sin\theta_{k}},\cdots ,e^{j(N-1)\pi \sin\theta_{k}}{]}^{T}\in {\mathbb{C}}^{N\times 1}$$

Then, the received data at the receiving array can be described as [11]

(8) $$X(t)=\sum_{k=1}^{K}\beta_{k}e^{j2\pi f_{pk}t}b(\theta_{k})a^{T}(r_{k},\theta_{k})\left[\begin{array}{c} s_{1}(t)\\ \vdots \\ s_{M}(t) \end{array}\right]+n(t)$$

where *β_k_* and *f_pk_* represent the amplitude and Doppler frequency of the *k*-th target, respectively. *n*(*t*) represents an *N* × 1 complex Gaussian white noise vector with zero mean. According to Equation (3), the received data after the matched filtering is expressed as [37]

(9) Y(t)=CH(t)+N(t)

(10) $$C=[c_{1}(r_{1},\theta_{1}),\cdots ,c_{K}(r_{K},\theta_{K})]=[a(r_{1},\theta_{1})⊗b(\theta_{1}),\cdots ,a(r_{K},\theta_{K})⊗b(\theta_{K})]$$

(11) $$H(t)=\sqrt{\frac{E}{M}}{\left[\beta_{1}e^{j2\pi f_{p1}t},\beta_{2}e^{j2\pi f_{p2}t},\ldots ,\beta_{k}e^{j2\pi f_{pk}t}\right]}^{T}$$

where *C* is the joint transmit-receive steering matrix, and ⊗ denotes the Kronecker product. *H*(*t*) is the signal matrix after matched filters. *N*(*t*) represents the noise vector after matching filter with the transmitted signal, and the noise covariance matrix is *σ*^2^*I_MN_*, where *σ*^2^ and *I_MN_* denote the noise variance and *M* × *N* identity matrix, respectively.

## 3. Unitary ESPRIT Algorithm for Ange and Range Estimation

### 3.1. Rotation Invariance of Subarrays

**Figure 2:**
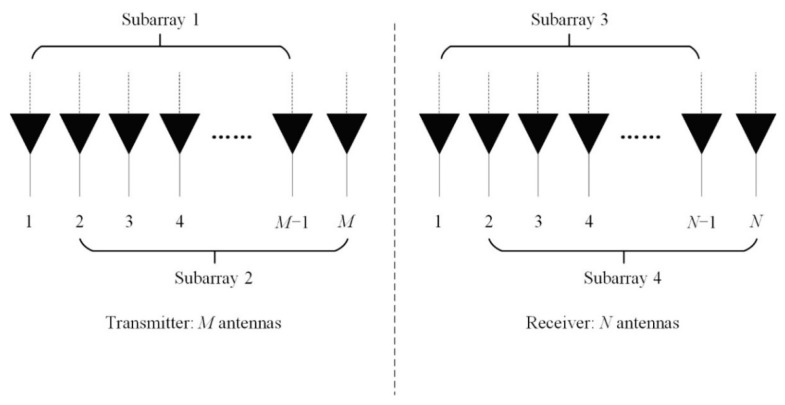
The division of the transmitting array and receiving array.

In this section, the complex-valued rotation invariance relation is introduced with respect to the transmitting array and receiving array, respectively. As shown in Figure 2, the transmitting array and receiving array of monostatic FDA-MIMO radar are divided into two overlapping subarrays,. Assuming that the two adjacent subarrays are identical, there exists a rotation invariance between Subarray 1 and Subarray 2, or Subarray 3 and Subarray 4. According to Equation (4), due to the existence of a coupling relationship between DOA and the range, it is necessary to obtain DOA information by the rotation invariance relationship of the receiving subarrays, and substitute the estimated DOA into the rotation invariance of transmitting arrays to get the range information. The complex-valued invariance relationship of the Subarray 3 and Subarray 4 can be expressed as [42]

(12) $$J_{2}b(\theta_{k})=e^{j\pi \sin\theta_{k}}J_{1}b(\theta_{k})$$

where $J_{1}=[\begin{array}{cc} I_{N-1} & 0_{(N-1)\times 1} \end{array}]$ and $J_{2}=[\begin{array}{cc} 0_{(N-1)\times 1} & I_{N-1} \end{array}]$ are selection matrices, and *0_w_* denotes the *w* × *w* null matrix. The functions of *J*_1_ and *J*_2_ are to select the first and last *N* − 1 rows of a matrix, respectively. Based on Equation (12), the invariance relationship in the joint steering vector can be expressed as

(13) $$\left(I_{M}⊗J_{2})c(r_{k},\theta_{k})=e^{j\pi \sin\theta_{k}}(I_{M}⊗J_{1})c(r_{k},\theta_{k}\right)$$

Under the assumption of *K* targets, the rotation invariance of each target can be written into a matrix form

(14) $$(I_{M}⊗J_{2})C=(I_{M}⊗J_{1})C\Xi_{R}$$

(15) $$\Xi_{R}=\left[\begin{array}{ccc} e^{j\pi \sin\theta_{1}} & & \\ & \ddots & \\ & & e^{j\pi \sin\theta_{k}} \end{array}\right]$$

where $\Xi_{R}$ contains the angle information of all targets. It can be shown that the columns in *C* span the same signal subspace as the column vectors in the signal subspace *E_s_* [43], we can obtain the following relationship

(16) $$E_{S}=C\Theta$$

where *E_s_* is composed of the *K* eigenvectors corresponding to the largest *K* eigenvalues of the covariance matrix of *Y*, and Θ is a non-singular matrix. Substituting Equation (16) into Equation (14), we can obtain the relationship between signal space of the subarrays

(17) $$(I_{M}⊗J_{2})E_{S}=(I_{M}⊗J_{1})E_{S}\Psi_{R}$$

where $\Psi_{R}=\Theta^{-1}\Xi_{R}\Theta$. It can be noticed that the diagonal elements of $\Xi_{R}$ are the eigenvalues of Ψ*_R_*.

Next, the complex-valued rotation invariance between Subarray 1 and Subarray 2 is considered to estimate range. The transmitting steering vectors of Subarray 1 and Subarray 2 satisfy the following equation

(18) $$J_{4}a(r_{k},\theta_{k})=e^{j(\pi \sin\theta_{k}-4\pi \frac{\Delta f}{c}r_{k})}J_{3}a(r_{k},\theta_{k})$$

where $J_{3}=[\begin{array}{cc} I_{M-1} & 0_{(m-1)\times 1} \end{array}]$ and $J_{4}=[\begin{array}{cc} 0_{(m-1)\times 1} & I_{M-1} \end{array}]$ stand for selection matrices to select the first and last *M* − 1 rows, respectively. For *K* targets, this relationship is extended to the joint steering vector which can be expressed as

(19) $$(I_{N}⊗J_{4})C=(I_{N}⊗J_{3})C\Xi_{T}$$

(20) $$\Xi_{T}=\left[\begin{array}{ccc} e^{j(\pi \sin\theta_{1}-4\pi \frac{\Delta f}{c}r_{1})} & & \\ & \ddots & \\ & & e^{j(\pi \sin\theta_{K}-4\pi \frac{\Delta f}{c}r_{K})} \end{array}\right]$$

where $\Xi_{T}$ contains the ranges of all the targets. According to Equation (16), Equation (19) can be rewritten as

(21) $$(I_{N}⊗J_{4})E_{S}=(I_{N}⊗J_{3})E_{S}\Psi_{T}$$

where $\Psi_{T}=\Theta^{-1}\Xi_{T}\Theta$. Because the calculation of the covariance matrix of *Y* and the acquisition of *E_S_* are based on complex-valued data, DOAs and ranges are estimated with relatively high complexity. Hence, based on the idea of a unitary ESPRIT algorithm, a novel unitary ESPRIT algorithm is proposed to reduce complexity and improve estimation accuracy.

### 3.2. Unitary ESPRIT in FDA-MIMO Radar

In this section, a complex-valued invariance is transformed into a real-valued invariance, and the DOAs and ranges are estimated by using the unitary ESPRIT algorithm. As there is no central Hermitian symmetric characteristic in *Y*, an extended receiving data matrix with the symmetric structure is defined as [44,45]

(22) $$Z=\left[\begin{array}{cc} Y & \Pi_{\mathrm{MN}}Y^{*} \end{array}\Pi_{L}\right]$$

where Π*_MN_* is an *M* × *N* exchange matrix with ones on its anti-diagonal and zeros elsewhere. Over the construction of Equation (22), *Z* is a generalized Centro-Hermitian matrix. The complex matrix *Z* is transformed into the real-valued matrix *Γ* by utilizing the unitary transformation. It can be expressed as [43]

(23) $$\Gamma =Q_{MN}^{H}ZQ_{2L}=Q_{MN}^{H}\left[\begin{array}{cc} Y & \Pi_{\mathrm{MN}}Y^{*} \end{array}\Pi_{L}\right]Q_{2L}$$

where (.)^H^ represents the conjugate transpose operator, and the sparse unitary matrix *Q_w_* is defined as

(24) $$\{\begin{array}{c} Q_{w}=\frac{1}{\sqrt{2}}\left[\begin{array}{cc} I_{w} & jI_{w}\\ \Pi_{w} & -j\Pi_{w} \end{array}\right]\\ Q_{w}=\frac{1}{\sqrt{2}}\left[\begin{array}{ccc} I_{w} & 0 & jI_{w}\\ 0^{T} & \sqrt{2} & 0^{T}\\ \Pi_{w} & 0 & -j\Pi_{w} \end{array}\right] \end{array}\begin{array}{ccc} & & \begin{array}{c} w\;\mathrm{is}\;\mathrm{even}\\ w\;\mathrm{is}\;\mathrm{odd} \end{array} \end{array}$$

Compared with Equation (9), Equation (22) is competent in doubling the number of snapshots. Then, the real-valued covariance *R*_Γ_ of the extended received data can be acquired by using the maximum likelihood estimation

(25) $$\begin{array}{l} R_{\Gamma }=\;\frac{1}{2L}\Gamma \Gamma^{H}\\ =\frac{1}{2}\left(Q_{MN}^{H}R_{Z}Q_{MN}\right)\\ =\frac{1}{2}\left[Q_{MN}^{H}\left(R_{Y}+\Pi_{MN}R_{Y}^{*}\Pi_{MN}\right)Q_{MN}\right] \end{array}$$

where *R_Y_* and *R_Z_* are the covariance calculated by *Y* and *Z*, respectively. The signal subspace ${\hat{E}}_{S}$ corresponds to *K* eigenvectors of large eigenvalues of *R*_Γ_. The remaining *MN* − *K* eigenvectors of small eigenvalues can obtain the noise subspace ${\hat{E}}_{N}$. Hence, ${\hat{E}}_{S}$ and ${\hat{E}}_{N}$ are both real-valued. Due to the unitary transformation in Equation (23), the complex-valued invariance relation in Equation (13) is transformed into real-valued invariance relation as follows

(26) $$K_{2}d_{k}=\tan\left(\frac{\pi \sin\theta_{k}}{2}\right)K_{1}d_{k}$$

(27) K1=Re{Q(N−1)mH(IM⊗J2)QMN}

(28) K2=Im{Q(N−1)mH(IM⊗J2)QMN}

where $d_{k}=Q_{MN}^{H}c_{k}$ is the real-valued steering vector. Re{.} and Im{.} denote the real and imaginary parts of a complex number, respectively. Considering *K*-independent targets, Equation (26) is expressed as

(29) $$K_{2}D=K_{1}D\Phi_{R}$$

(30) $$\Phi_{R}=\left[\begin{array}{ccc} \tan(\frac{\pi \sin\theta_{1}}{2}) & & \\ & \ddots & \\ & & \tan(\frac{\pi \sin\theta_{k}}{2}) \end{array}\right]$$

where *D* = [*d*_1_, *d*_2_, …, *d_k_*], and Φ*_R_* is a real diagonal matrix whose diagonal elements contain the desired angle information. According to Equation (16), Equation (29) can be rewritten as

(31) $$K_{2}{\hat{E}}_{S}=K_{1}{\hat{E}}_{S}\Sigma_{R}$$

where $\Sigma_{R}=\Theta_{R}^{-1}\Phi_{R}\Theta_{R}$. $\Theta_{R}$ is the left eigenvector matrix of $\Sigma_{R}$. By using the total least squares (TLS) method to solve Equation (31), DOA can be estimated as follows

(32) $${\hat{\theta }}_{k}=\arcsin\left(\frac{2\arctan{\left[(\Phi_{R})\right]}_{k}}{\pi }\right)$$

Similarly, the rotation invariance between Subarray 1 and Subarray 2 can be transformed into

(33) $$K_{4}d_{k}=\tan\left(\frac{\pi (\sin\theta_{k}-4\Delta f\frac{r_{k}}{c})}{2}\right)K_{3}d_{k}$$

(34) K3=Re{Q(m−1)NH(IN⊗J4)QMN}

(35) K4=Im{Q(m−1)NH(IN⊗J4)QMN}

For *K* targets, Equation (33) can be integrated into matrix form

(36) $$K_{4}D=K_{3}D\Phi_{T}$$

(37) $$\Phi_{T}=\left[\begin{array}{ccc} \tan(\frac{\pi \sin\theta_{1}-4\pi \Delta f\frac{r_{1}}{c}}{2}) & & \\ & \ddots & \\ & & \tan(\frac{\pi \sin\theta_{k}-4\pi \Delta f\frac{r_{k}}{c}}{2}) \end{array}\right]$$

where Φ*_T_* is a real diagonal matrix whose diagonal elements contain the desired range of information. In the same way as Equation (31), we can obtain the rotational invariance of the signal subspace

(38) $$K_{4}{\hat{E}}_{S}=K_{3}{\hat{E}}_{S}\Sigma_{T}$$

where $\Sigma_{T}=\Theta_{T}^{-1}\Phi_{T}\Theta_{T}$. $\Theta_{T}$ is the inverse of the left eigenvector matrix of $\Sigma_{T}$. Substituting Equation (32) into Equation (37), the range estimation is solved with the TLS method.

(39) $${\hat{r}}_{k}=\frac{2\arctan{\left[(\Phi_{R})\right]}_{k}-2\arctan{\left[(\Phi_{T})\right]}_{k}}{4\pi \Delta f}c$$

Due to the correlation of Φ*_R_* and Φ*_T_* in Equation (39), the ranges will be miscalculated without the pairing of Φ*_R_* and Φ*_T_*. Hence, we employ an automatic pairing method to implement correct range estimation.

### 3.3. The Pairing of DOAs and Ranges

In this section, we analyze the speciality of $\Theta_{T}$ and $\Theta_{R}$, and introduce the means to achieve pairing. $\Theta_{T}$ and $\Theta_{R}$ are eigenvectors of $\Sigma_{T}$ and $\Sigma_{R}$, respectively. Since $\Sigma_{T}$ and $\Sigma_{R}$ are calculated by ${\hat{E}}_{S}$, there must be a random row of $\Theta_{R}$ identical to a specific row of $\Theta_{T}$. Supposing that all of the *K* targets are independent, we notice that any two rows of $\Theta_{R}$ are orthogonal because any two eigenvalues of $\Theta_{T}$ are different. In this paper, considering algorithm complexity, we obtain the automatic pairing of Φ*_T_* and Φ*_R_* by decomposing the $\Sigma_{T}+j\Sigma_{R}$, which can be expressed as

(40) $$\Sigma_{T}+j\Sigma_{R}=\Theta_{TR}^{-1}\{\Phi_{T}+j\Phi_{R}\}\Theta_{TR}$$

where $\Theta_{TR}$ is the left eigenvector matrix. Hence, Φ*_T_* and Φ*_R_* can be automatically paired by the eigenvector matrix $\Theta_{TR}$. Considering the periodic phase ambiguity problem, we take a step to distinguish the phase ambiguity before calculating ranges.

### 3.4. The Solution of Periodic Ambiguity of Transmitter

In this section, we analyze the periodic phase ambiguity and adopt an ambiguity judgment method to obtain the correct range estimation. Since the period of tan in Equation (26) is π, and sin*θ_k_* ∈ (−1, 1), DOAs can be estimated by Equation (32) without periodic ambiguity. However, there is phase ambiguity in range estimation due to sin*θ_k _*− 4Δ*fr_k_*/c ∈ (−3, 1) and *r_k_* ∈ (0, c/2Δ*f*). Therefore, *r_k_* obtained by Equation (39) is misestimated, because the tangent of π(sin*θ_k_* − 4Δ*fr_k_*/c)/2 is equal to the tangent of π(sin*θ_k_* − 4Δ*fr_k_*/*c*)/2 + π when sin*θ_k_* − 4Δ*fr_k_*/c ∈ (−3, −1). Note that π(sin*θ_k_* − 4Δ*fr_k_*/*c*)/2 < πsinθ*_k_*/2< π(sin*θ_k_* − 4Δ*fr_k_*/*c*)/2 + π. Since Φ*_T_* and *Φ_R_* are relevant to π(sin*θ_k_* − 4Δ*fr_k_*/*c*)/2 and πsinθ*_k_*/2, respectively, we determine the range of π(sin*θ_k_* − 4Δ*fr_k_*/c)/2 by comparing arctan[Φ*_T_*]*_k_* and arctan[*Φ_R_*]*_k_*. If arctan[Φ*_T_*]*_k_* > arctan[(*Φ_R_*)]*_k_*, due to arctan[Φ*_T_*]*_k_* ∈ (−π/2, π/2), there is a phase ambiguity, as π(sin*θ_k_* − 4Δ*fr_k_*/c)/2 is considered to lie in (−π/2, π/2) when π(sin*θ_k_* − 4Δ*fr_k_*/c)/2 ∈ (−3π/2, −π/2). We can use a phase shift π to solve the periodic phase ambiguity problem. Hence, the true phase value of Φ*_T_* can be calculated as

(41) $$\frac{\pi }{2}(\sin{\hat{\theta }}_{k}-4\Delta fr_{k}/c)=\arctan(\Phi_{T})-\pi$$

*r_k_* can be given by

(42) $${\hat{r}}_{k}=\frac{\arctan{\left[(\Phi_{R})\right]}_{k}-\arctan{\left[(\Phi_{T})\right]}_{k}+\pi }{2\pi \Delta f}c$$

Otherwise, there is no phase ambiguity and the true phase value of Φ*_T_* can be given by

(43) $$\frac{\pi }{2}(\sin{\hat{\theta }}_{k}-4\Delta fr_{k}/c)=\arctan(\Phi_{T})$$

*r_k_* can be given by

(44) $${\hat{r}}_{k}=\frac{\arctan{\left[(\Phi_{R})\right]}_{k}-\arctan{\left[(\Phi_{T})\right]}_{k}}{2\pi \Delta f}c$$

The main steps of the proposed algorithm are summarized in Algorithm 1.
**Algorithm 1:** A Novel Unitary ESPRIT for Monostatic FDA-MIMO Radar1:Construct the extended received data matrix *Z* via (22).2:Take the unitary transformation using (23) and obtain *R*_Γ_ via (25).3:Perform eigenvalue decomposition of *R*_Γ_ and return ${\hat{E}}_{S}$.4:Calculate $\Sigma_{T}$ and $\Sigma_{R}$ via (31) and (38), respectively.5:Obtain the automatically paired Φ*_T_* and Φ*_R_* via (40).6:Compute the DOAs by (32) and ranges by (42) or (44).

## 4. CRB and Complexity Analysis

### 4.1. CRB

In this section, we analyze the CRBs of angle and range for the monostatic FDA-MIMO radar. According to Equation (9), the concrete expression of *R_Y_* is written as

(45) $$R_{Y}=\frac{1}{L}YY^{H}=CR_{H}C^{H}+\sigma^{2}I$$

where $R_{H}$ is the covariance of *H* in Equation (9), and *L* is the number of snapshots, and *σ*^2^ denotes the noise power. Under the assumption of *K* targets, the unknown parameter to be estimated is

(46) $$\eta ={\left[\theta^{T},r^{T}\right]}^{T}={\left[\theta_{1},\ldots ,\theta_{k},r_{1},\ldots ,r_{k}\right]}^{T}$$

Then, the Fisher information matrix (FIM), with respect to *η*, is [41]

(47) $$F=\left[\begin{array}{cc} F_{\theta \theta } & F_{\theta r}\\ F_{r\theta } & F_{\mathrm{rr}} \end{array}\right]$$

The expression of every block of *F* can be written as

(48) Fθθ=2Lσ2Re{(CθHΠC⊥Cθ)⊙(RHCHRY−1CRH)T}

(49) Fθr=2Lσ2Re{(CθHΠC⊥Cr)⊙(RHCHRY−1CRH)T}

(50) Frθ=2Lσ2Re{(CrHΠC⊥Cθ)⊙(RHCHRY−1CRH)T}

(51) Frr=2Lσ2Re{(CrHΠC⊥Cr)⊙(RHCHRY−1CRH)T}

where *C_θ_* and *C_r_* are partial derivations of *C* with respect to *θ* and *r*, and $\Pi_{C}^{⊥}=I-C{\left(C^{H}C\right)}^{-1}C^{H}$. The *C_θ_* and *C_r_* can be written as

(52) $$C_{\theta }=\left[c_{1\theta },c_{2\theta },\ldots ,c_{K\theta }\right]$$

(53) $$C_{r}=\left[c_{1r},c_{2r},\ldots ,c_{\mathrm{Kr}}\right]$$

where

(54) $$c_{k\theta }=\frac{\partial c(\theta_{k},r_{k})}{\partial \theta_{k}}=\frac{\partial a(\theta_{k},r_{k})}{\partial \theta_{k}}⊗b(\theta_{k})+a(\theta_{k},r_{k})⊗\frac{\partial b(\theta_{k})}{\partial \theta_{k}}k=1,2,\ldots K$$

(55) $$c_{kr}=\frac{\partial c(\theta_{k},r_{k})}{\partial r_{k}}=\frac{\partial a(\theta_{k},r_{k})}{\partial r_{k}}⊗b(\theta_{k}),k=1,2,\ldots K$$

We derive part of Equations (54) and (55) as

(56) $$\frac{\partial a(\theta_{k},r_{k})}{\partial \theta_{k}}=j\pi \cos(\theta_{k})\left[\begin{array}{ccc} 0 & & \\ & \ddots & \\ & & M-1 \end{array}\right]a(\theta_{k},r_{k})$$

(57) $$\frac{\partial b(\theta_{k})}{\partial \theta_{k}}=j\pi \cos(\theta_{k})\left[\begin{array}{ccc} 0 & & \\ & \ddots & \\ & & N-1 \end{array}\right]b(\theta_{k})$$

and

(58) $$\frac{\partial a(\theta_{k},r_{k})}{\partial r_{k}}=-j4\pi \frac{\Delta f}{c}\left[\begin{array}{ccc} 0 & & \\ & \ddots & \\ & & M-1 \end{array}\right]a(\theta_{k},r_{k})$$

Then, every block of *F* is determined by Equations (48)–(51). Then, the CRB matrix can be obtained by

(59) CRB=F−1=σ22LRe{(WHΠC⊥W)⊙PT}−1

where $W=[C_{\theta }\;C_{r}]$, $P=\left[\begin{array}{cc} P_{1} & P_{2}\\ P_{3} & P_{4} \end{array}\right]$ and $P_{1}=P_{2}=P_{3}=P_{4}=R_{H}C^{H}R_{Y}^{-1}CR_{H}$.

### 4.2. Complexity

To compare the presented algorithm and the ESPRIT algorithm of [42], the specific analysis of the computational complexity is provided. In this paper, we transform received data from the complex domain to the real domain by a unitary transformation. Hence, the calculation of eigenvalue decomposition and generalized inverse depend on the real domain. The calculation of a complex product is equivalent to the calculation of four real products. The concentration of computational complexity in the presented algorithm is based on calculating the covariance matrix, utilizing the eigenvalue decomposition, obtaining signal subspace, solving the solution for angle and range, and achieving pairing for angle and range. The calculation of *R*_Γ_ needs *O*{2*L*(*MN*)^2^} flops, where *M* and *N* denote the number of transmitting and receiving array elements, respectively, and *L* is the number of snapshots. The eigenvalue decomposition of *R*_Γ_, to obtain the signal subspace and the noise subspace, needs *O*{(*MN*)^3^} flops. The complexity required to solve for $\Sigma_{R}$ is *O*{*M*(*N* − 1)(2*K*)^2^}, where *K* is the number of targets. Similarly, solving $\Sigma_{T}$ needs *O*{*N*(*M* − 1)(2*K*)^2^} flops. The eigenvalue decomposition in Equation (40) and the pairing of angle and range need *O*{4(2*K*)^3^ + 2*K*^3^}. Here, we ignore the complexity of solving periodic ambiguity steps because they are too small. Thus, the complexity of the proposed algorithm is

(60) $$O\left\{2L{\left(MN\right)}^{2}+{\left(MN\right)}^{3}+4K^{2}(2MN-m-N)+34K^{3}\right\}$$

In [42], the complexity of the ESPRIT algorithm for estimation of angle and range is

(61) $$O\left\{2L{\left(MN\right)}^{2}+{\left(2MN\right)}^{3}+4K^{2}(5MN-2m-2N)+31K^{3}\right\}$$

By the comparison of Equations (60) and (61), the computational complexity of the proposed algorithm is much lower than [42]. Furthermore, later in the simulation, we give the comparison results regarding complexity.

## 5. Simulation Results

In this section, we provide several simulation results to evaluate the performance of the proposed algorithm for angle and range estimation in monostatic FDA-MIMO radar with ULA. The ESPRIT algorithm in the same model is chosen for comparison [42]. In all simulations, assume that the reference carrier frequency *f*_0_, namely the minimum frequency, is 3 GHz, and the frequency increment Δ*f* is 10^3^ Hz. According to the relationship of Equation (1), the maximum frequency and the number of bins depend on the number of transmitting arrays. The noise is assumed to be the uniform complex white Gaussian noise. The reflection coefficient of the target is set to 1. The number of Monte Carlo experiments is set to 500.

### 5.1. Estimated Results

**Figure 3:**
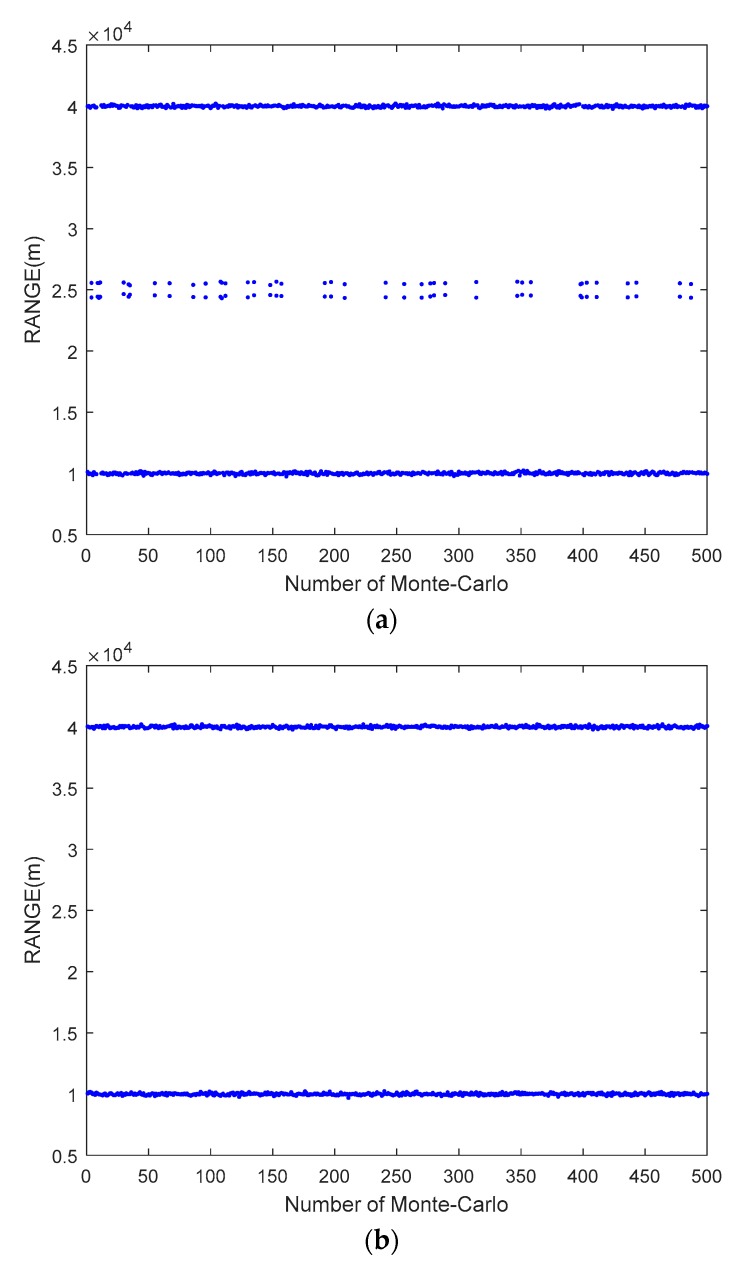
The range estimation results obtained by the proposed algorithm with unpairing and pairing. (**a**) Unpaired range estimation; (**b**) paired range estimation.

**Figure 4:**
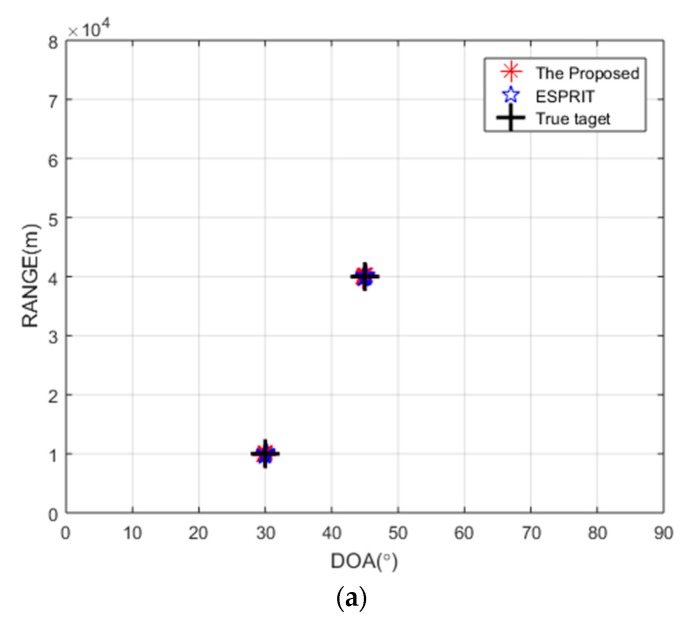

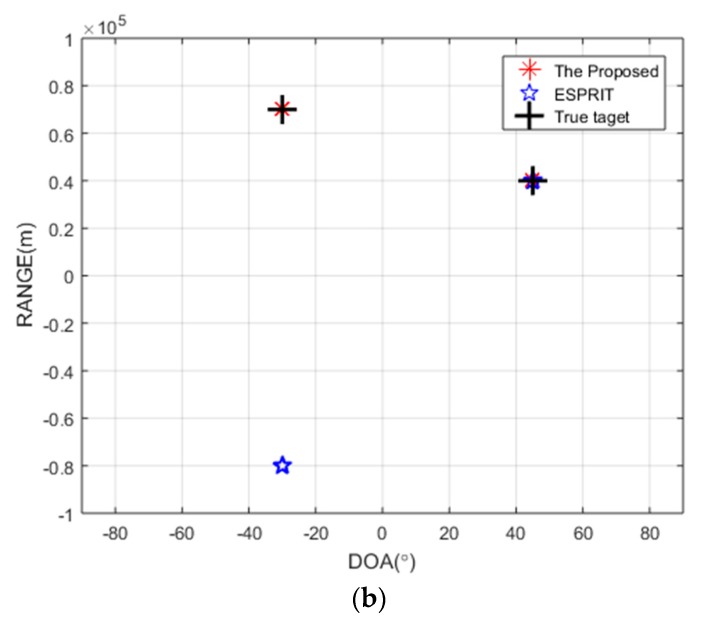
Location of the target under different target parameters by the proposed algorithm and ESPRIT algorithm. (**a**) Targets without phase ambiguity; (**b**) targets with phase ambiguity.

In this section, the SNR is set to 10 dB, and the number of snapshots is 50, and the number of transmitting array elements *M* and receiving array elements *N* are both set to eight. Figure 3a,b shows the unpaired and paired estimation of range, respectively, obtained by the proposed algorithm, where the two-dimensional parameters of the target are set to (45°, 40 km) and (30°, 10 km). It is noted that an incorrect range estimation is shown in Figure 3a, which is caused by the mismatch between the eigenvalues of $\Sigma_{R}$ and $\Sigma_{T}$. It is seen in Figure 3b that the pairing method can obtain the correct range estimation. Figure 4a,b shows the estimation results of angle and range acquired by the proposed algorithm and the ESPRIT algorithm, where the targets in Figure 4a are the same as those of Figure 3, and the targets in Figure 4b are assumed to be (45°, 40 km) and (−30°, 70 km). As there is no period ambiguity for (45°, 40 km) and (30°, 10 km), the proposed algorithm and the ESPRIT algorithm can both obtain accurate estimation, as shown in Figure 4a. It is shown in Figure 4b that the proposed algorithm can effectively solve the period ambiguity, because (−30°, 70 km) satisfies sin*θ_k_* − 4Δ*fr_k_*/*c* ∈ (−3, −1).

### 5.2. RMSE Versus SNR

**Figure 5:**
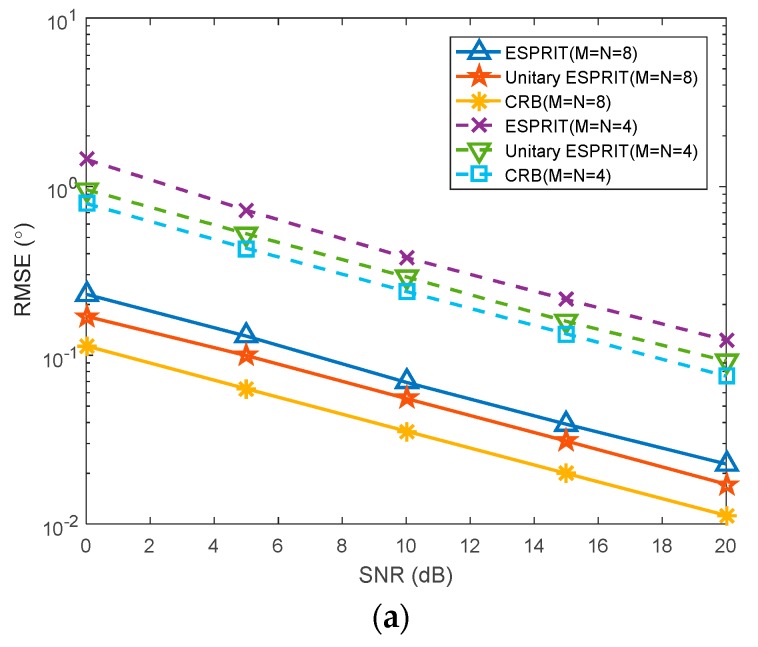

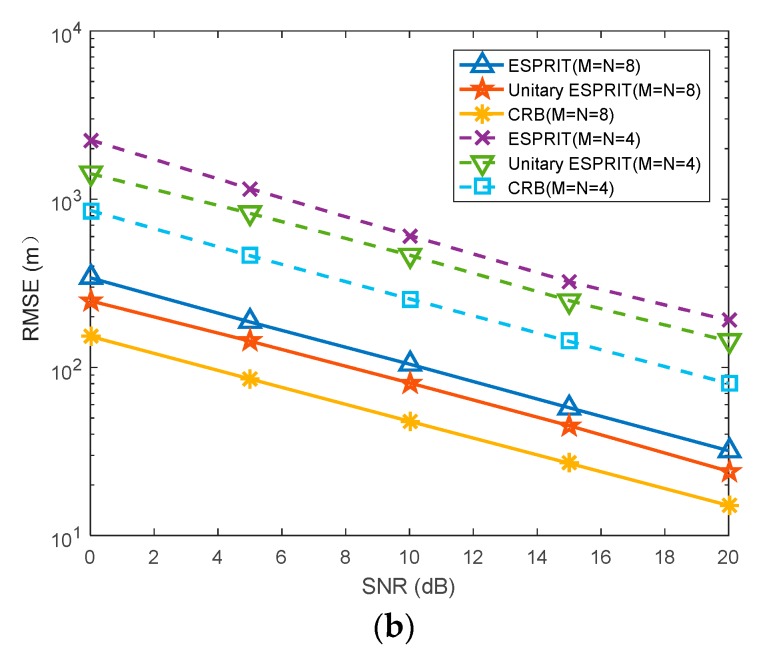
Estimation of target parameters by the proposed algorithm and ESPRIT algorithm with different SNR. (**a**) RMSE of DOA versus SNR; (**b**) RMSE of range versus SNR.

In this section, the target is set to (45°, 40 km) and (30°, 10 km), and the number of snapshots is 50. The number of transmitting array elements *M* and receiving array elements *N* are set to *M* = *N* = 4 and *M* = *N* = 8, respectively. The SNR increases from 0 dB to 20 dB, with each step being 2 dB. Figure 5a,b shows the root mean square errors (RMSEs) of the proposed algorithm and the ESPRIT algorithm with the different SNR. Meanwhile, the CRBs of angle and range in the monostatic FDA-MIMO radar are chosen for the assessment of the performance of the proposed algorithm. The RMSEs of angle and range are respectively defined as

(62) $$RMSE_{\theta }=\sqrt{\frac{1}{G}\frac{1}{K}\sum_{g=1}^{G}\sum_{k=1}^{K}{\left(\theta_{k}-{\hat{\theta }}_{k}\right)}^{2}}$$

(63) $$RMSE_{r}=\sqrt{\frac{1}{G}\frac{1}{K}\sum_{g=1}^{G}\sum_{k=1}^{K}{\left(r_{k}-{\hat{r}}_{k}\right)}^{2}}$$

where *G* represents the number of Monte Carlo experiments. We can observe that the RMSEs of the proposed algorithm are closer to the CRBs. This indicates that the performance of the proposed method is better than the ESPRIT algorithm with the identical SNR.

### 5.3. RMSE Versus Number of Snapshots

**Figure 6:**
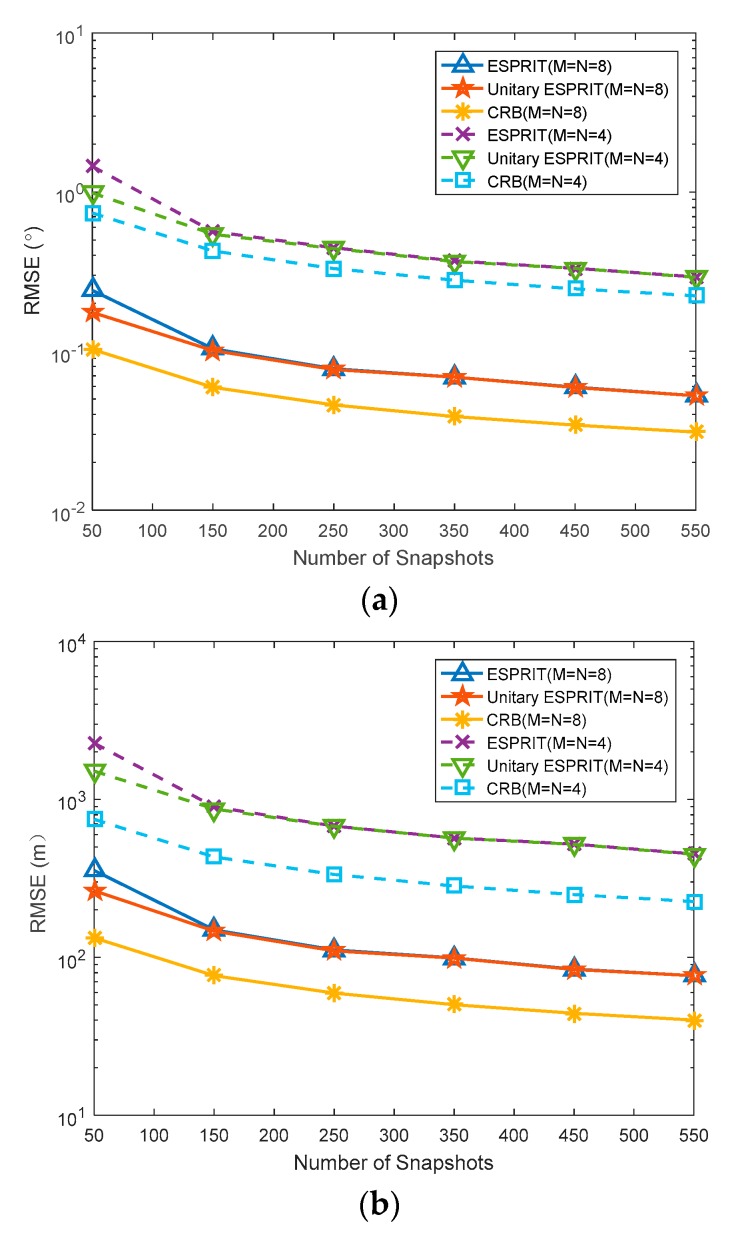
Estimation of target parameters by the proposed algorithm and ESPRIT algorithm with different SNR. (**a**) RMSE of DOA versus SNR; (**b**) RMSE of range versus SNR.

In the simulation, the target is set to (45°, 40 km) and (30°, 10 km), and the SNR is 0 dB. The number of transmitting array elements *M* and receiving array elements *N* are both set to eight and four, respectively. We set the initial number of snapshots to be 50, and observe the effect of the number of snapshots on the RMSEs by intervals of 100. Figure 6a, b shows the RMSEs of angle and range versus the number of snapshots, respectively. This indicates that the performance of the proposed algorithm is better than that of the ESPRIT algorithm with the same number of snapshots.

### 5.4. Computational Complexity

**Figure 7:**
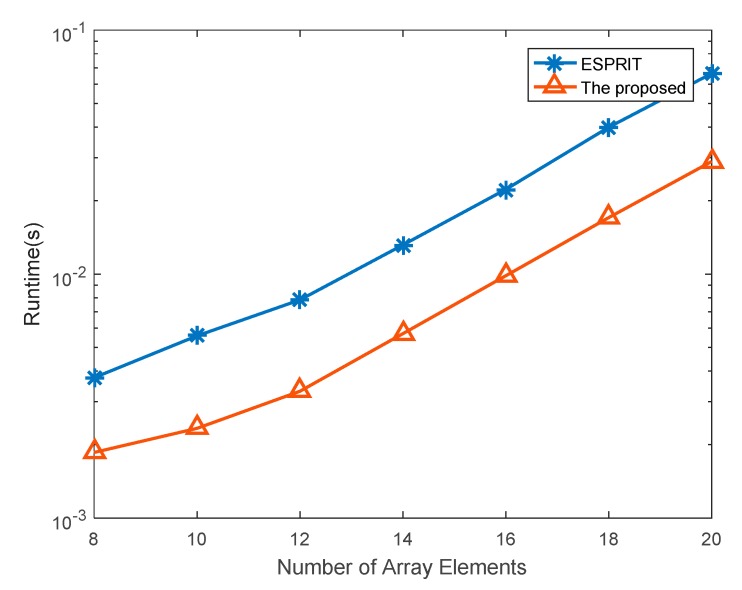
The runtime of the ESPRIT algorithm and the proposed algorithm with the different number of array elements.

In this part, the runtime of the proposed algorithm is compared with that of the ESPRIT algorithm. The target is set to (45°, 40 km), and (30°, 10 km), the SNR is set to 0 dB, and the number of snapshots is 50. The number of transmitting array elements is equal to that of the receiving array elements, i.e., *M* = *N*, and the transmitting array number *M* is changed in this simulation. The required runtime of the two algorithms is shown in Figure 7. The runtime of the proposed algorithm is less than that of the ESPRIT algorithm.

We can summarize this with two situations, according to the existence of periodic ambiguity. In the case of periodic ambiguity, the ESPRIT algorithm cannot obtain the correct estimation of target parameters, but the proposed algorithm can accurately get the angles and ranges of the target. In the absence of periodic ambiguity, the proposed algorithm and ESPRIT algorithm can both obtain a correct estimation of target parameters. The estimation accuracy of the proposed algorithm is higher than that of the ESPRIT algorithm, and the running time is shorter than that of the ESPRIT algorithm. Due to the extended receiving data and the unitary transformation operation of the proposed algorithm, the number of snapshots is as twice as the original number, and the complex data is transformed into the real data, which greatly reduces the computational complexity.

## 6. Conclusions

In this paper, a novel unitary ESPRIT algorithm is proposed for the angle and range estimation in a monostatic FDA-MIMO radar. In the proposed method, the angle and range are estimated by using the rotation invariance between the specific subarrays. Then, we make a specific analysis of periodic ambiguity and propose a method to solve that. Additionally, the computational complexity of the proposed algorithm is compared with that of the ESPRIT algorithm. The theoretical performance of the proposed algorithm is verified by computer simulation. In future work, we will focus on how to estimate the parameters of targets when mutual coupling errors exist in the FDA-MIMO radar, how to use the proposed algorithm in more general array structures, and how to use the proposed algorithm to estimate parameters in a colored noise environment.
